# Multi‐level analysis of the learning health system: Integrating contributions from research on organizations and implementation

**DOI:** 10.1002/lrh2.10226

**Published:** 2020-04-02

**Authors:** Michael I. Harrison, Stephen M. Shortell

**Affiliations:** ^1^ Senior Social Scientist Agency for Healthcare Research and Quality Rockville Maryland USA; ^2^ Professor of the Graduate School, Blue Cross of California Distinguished Professor of Health Policy and Management, Emeritus; Professor of Organization Behavior, Emeritus School of Public Health and Haas School of Business, University of California ‐ Berkeley Berkeley California USA

**Keywords:** implementation science, learning health system, organizational learning

## Abstract

**Introduction:**

Organizations and systems that deliver health care may better adapt to rapid change in their environments by acting as learning organizations and learning health systems (LHSs). Despite widespread recognition that multilevel forces shape capacity for learning within care delivery organizations, there is no agreed‐on, comprehensive, multilevel framework to inform LHS research and practice.

**Methods:**

We develop such a framework, which can enhance both research on LHSs and practical steps toward their development. We draw on existing frameworks and research within organization and implementation science and synthesize contributions from three influential frameworks: the Consolidated Framework for Implementation Research, the social‐ecological framework, and the organizational change framework. These frameworks come, respectively, from the fields of implementation science, public health, and organization science.

**Results:**

Our proposed integrative framework includes both intraorganizational levels (individual, team, mid‐management, organization) and the operating and general environments in which delivery organizations operate. We stress the importance of examining interactions among influential factors both within and across system levels and focus on the effects of leadership, incentives, and culture. Additionally, we indicate that organizational learning depends substantially on internal and cross‐level alignment of these factors. We illustrate the contribution of our multilevel perspective by applying it to the analysis of three diverse implementation initiatives that aimed at specific care improvements and enduring system learning.

**Conclusions:**

The framework and perspective developed here can help investigators and practitioners broadly scan and then investigate forces influencing improvement and learning and may point to otherwise unnoticed interactions among influential factors. The framework can also be used as a planning tool by managers and practitioners.

## 1 INTRODUCTION

Health care organizations in the United States and other industrialized nations face pressures to contain costs, improve quality, reduce health inequalities, and care for an aging population.^1^, ^2^, ^3^ They also must adapt to major changes in payments and budgets; treatments and technologies; and patterns of ownership, organization, and delivery of care.^2^, ^4^, ^5^, ^6^, ^7^, ^8^

A growing group of health care leaders and researchers anticipate that delivery systems can adapt to these challenges by engaging in rapid learning; innovation; exploitation of emerging digital technologies; and development of enhanced capabilities in system redesign and quality improvement. This vision is captured in discussions of the learning health system (LHS)^9^, ^10^, ^11^, ^12^, ^13^ and learning organizations.^14^, ^15^, ^16^, ^17^, ^18^, ^19^ The National Academy of Medicine (NAM)^1^ defines the “Continuously Learning Health Care System” as “one in which science and informatics, patient‐clinician partnerships, incentives, and culture are aligned to promote and enable continuous and real‐time improvement in both the effectiveness and efficiency of care”(^13^, p. 17).

In this article, we seek to strengthen the emerging understanding of organizational and system learning in health care as resulting from interactions among diverse factors, which operate at multiple levels within and beyond individual care delivery organizations. In the view of the NAM and others,^15^, ^16^ LHS care organizations gain knowledge and improvement capacity as their members scan the environment for knowledge and innovations; select, try, and test them; learn from internal data and experience; and compare their findings with those of other care organizations. In the NAM's formulation, learning systems have the following characteristics:

Real‐time access to evidence; digital capture of the care experience, strong partnerships with patients, families, and other care givers; incentives aligned to promote continuous improvement and provision of high‐value care; full transparency; a leadership‐instilled culture of learning, and supportive processes such as team training and skill building, systems analysis, and feedback for continuous learning and system improvement. (Source^13^, p. 18, table S2)

Like many treatments of learning organizations, the NAM's LHS model characterizes learning in terms of observable, behavioral changes, rather than primarily cognitive ones.^20^ This behavioral view defines organizational learning as “the process of improving actions through better knowledge and understanding,” (^21^, p. 803). The LHS framework can be applied to individual health care organizations, delivery systems, organizational networks,^22^ and national and international health systems.^23^ In this article, we apply it to care organizations.

Research has examined how organizational learning and improvement are affected by internal organizational factors, such as individual training and learning to improve work while doing it^24^: teamwork, leadership, information technology, knowledge management, and culture.^18^, ^25^, ^26^, ^27^, ^28^, ^29^, ^30^, ^31^, ^32^, ^33^, ^34^ Studies also identify influences on learning within an organization's operating environment, including collaboration among care organizations^35^; partnerships with external researchers^36^; funding for research, innovation, and other activities supporting learning^29^; and payment for high value care and other forms of care improvement.^25^, ^29^

## 2 QUESTIONS OF INTEREST

It is widely recognized that the factors influencing organizational learning in health care and in other industries operate at multiple levels within and outside of care organizations.^28^, ^30^, ^37^, ^38^ It is particularly important to examine multiple levels of learning in health care because of the organizational complexity of care organizations and delivery systems and their dependencies on external agencies and conditions. Only a multilevel approach can adequately take account of the occupational diversity and interdependence of the work; interactions within professional hierarchies and between professionals and administrators; and the wide range of external influences and constraints on how work is accomplished.^39^ A multilevel perspective thus holds promise for advancing research that will provide actionable knowledge for health care organizations seeking to become learning healthcare systems.

Currently, there is no agreed‐on, comprehensive, multilevel framework for examining factors and processes shaping organization and system learning in health care. To address this limitation, we draw on organization and implementation science to develop a framework that can be used to advance the study of organizational learning in the healthcare sector.

## 3 METHODS

We first review three helpful multilevel frameworks from related fields. We then synthesize contributions from these frameworks and from research into an integrated framework showing factors influencing learning at different system levels. We illustrate the contribution of the multilevel perspective by applying it to the development of three initiatives that aimed at specific care improvements and enduring system learning. Next, we highlight the influence and dynamics of incentives, culture, and leadership, three fertile areas for research on cross‐level relations among factors affecting learning. We conclude by discussing some research and practice implications of our framework and the underlying multilevel perspective.

## 4 RESULTS

### 4.1 Three multilevel frameworks

A variety of studies and frameworks on dissemination, implementation, organizational change, and public health identify system levels at which influential factors operate.^40^, ^41^, ^42^, ^43^, ^44^ These levels range from the individual and work‐team to the environment of the focal organization or delivery system. Multilevel approaches often include lists of influential factors and acknowledge possible cross‐level relationships among them. Although the frameworks reviewed here were originally developed to address issues other than organizational learning in health care, they can contribute to LHS research.

#### 4.1.1 The Consolidated Framework for Implementation Research

The Consolidated Framework for Implementation Research (CFIR) is one of the most widely cited frameworks in Implementation Science.^40^, ^45^ It synthesizes findings from empirical studies and from 19 earlier conceptualizations and frameworks on knowledge transfer, implementation of evidence‐based practice, dissemination, and organizational change. The CFIR's research utility is growing, thanks to a community of researchers who are actively developing standard, validated measures for many of its constructs.^46^ The CFIR defines constructs for five domains: Intervention Characteristics; the Outer Setting (environment) of the organization in which implementation occurs; the Inner (organizational) Setting; involved Individuals; and the Implementation Process. Within the Inner Setting, the CFIR calls attention to the team, unit, and service levels, along with the organization as a whole.

The CFIR was developed for research on implementation of evidence‐based practices, but its multilevel perspective and many of its constructs have also been applied to research on collective learning and broad improvement programs. For example, The Veterans Health Administration uses the CFIR to guide and evaluate their efforts to become an effective learning health care system.^47^ The CFIR facilitates research on learning by including a construct for learning climate, defined as one in which leaders actively seek team members' inputs; team members feel that they are essential partners in the change process and feel psychologically safe to try new methods; and members have enough time and space for reflective thinking and evaluation. Additional contributions of the CFIR include its emphasis on interaction among the five domains and its distinction between formally appointed, internal implementation leaders and informal champions and opinion leaders.

Despite its contributions, there are limitations to applying the CFIR to LHS research and practice. The framework was developed and has mainly been used for studies of implementation of discrete evidence‐based practices, rather than broad organizational changes of the sort required for LHS development. The main constructs within the Outer Setting domain do not include market forces, knowledge, technology, social norms, and values, all of which may impact organizational learning. Nor do the Inner Setting domains cover some important organizational factors delineated by the NAM and other models of organizational learning,^18^, ^25^ including digital capture of the care experience, partnerships with patients, and supportive processes like systems engineering. The CFIR only cites information technology as an intervention characteristic and as a factor affecting access to knowledge about interventions. Additionally, the framework does not clearly distinguish the roles in change programs played by leaders at each organizational level.

#### 4.1.2 The social‐ecological framework

The social‐ecological perspective, which is widely used in practice and research on health behavior and public health,^44^, ^48^, ^49^ identifies multilevel determinants of health behavior. Based on this perspective, Tabak et al^43^ provide a socio‐ecological framework that distinguishes between the levels of the individual, organization (including hospitals, service organizations, and places of employment), community (local government, neighborhood), and system (eg, hospital system and government/policy).

Advantages of extending this framework to LHS research include its explicit assumptions that influential factors interact with one another within and across levels^44^, ^49^ and that some external “community” factors exercise immediate influences on care practice, while other external factors (at the “system” level) have less immediate and more general effects. Some community factors that affect care, such as the services available in a community and the needs and capacities of its members (eg, for self‐management and interaction with health providers), may also affect organizational learning.^19^

Although helpful, the socio‐ecological framework concentrates on programs and factors affecting individual health behavior and hence is less readily adaptable to investigations of learning and LHS operations than the CFIR. Additionally, the promising distinction between the community and system levels does not adequately fit the full range of factors affecting organizational learning. For example, care delivery organizations in a learning collaborative may directly influence one another's learning. However, these organizations are not necessarily located in the immediate, surrounding “community.”

Instead of trying to fit the socio‐ecological terminology to learning, it seems more helpful to follow the organizational literature, which distinguishes conditions that directly impact operations (sometimes called the “task” or “close” environment^50^) from a set of “general” environmental conditions, that have less immediate effects. The immediate, operating environment includes interorganizational cooperation and competition, regulations, and sources of payment and revenue. The more distant, general environment incudes scientific and technological developments, socio‐economic, and political conditions.

#### 4.1.3 Organizational change framework

Adding a third framework,^51^ which is derived from research on organizational change in health care, can help overcome some of the gaps that emerge when we try to apply the previous two frameworks to organizational and system‐wide learning in health care. Ferlie and Shortell originally applied their framework to initiatives to improve care quality across entire care organizations or delivery systems. The authors identify four main levels affecting such major organizational changes: the individual, group or team, overall organization, and larger system or environment. The system/environment level includes the political economy and markets for health care, along with institutional forces, such as regulatory and payment bodies and shared information about organizational practices and performance. At each level, “core properties” of leadership, culture, team development, and information technology influence organizational change and are influenced by it. When these properties are aligned and supportive of organization‐level learning and improvement, they closely resemble the LHS characteristics articulated by the NAM^13^ and others. Similarly, the core properties of this model capture features of many of the intraorganizational “building blocks” for organizational learning identified in the research‐based model of learning developed by Singer et al.^18^

The organizational change framework contains important implications for research on organization‐level learning. Individual learning must be communicated and managed for it to contribute to learning by other staff members, teams, or the entire organization. Similarly, teams can provide input into higher level learning when they implement evidence‐based practices^52^ or engage in quality improvement.^37^ Organization‐level learning synthesizes learning at these lower levels and applies the resulting knowledge to achieving strategic priorities and organizational goals.

Another contribution of the organizational change framework is its focus on effects of the extra‐organizational environment, including external incentives and other organizations. The authors' distinction between competitive and cooperative interorganizational relations contains important implications for learning. Competition may discourage innovative organizations from sharing knowledge and beneficial practices with one another. A further contribution of this framework is its recognition that entire organizations undergo changes, as well as serving as contexts for internal learning and for targeted implementation and improvement. Just as planned change can shape LHS capacities, so may unintended organizational changes, such as those flowing from leadership transitions, internal power struggles, external mandates, adaptation to external developments, and environmental selection.^53^

The organizational change framework underlines the importance of information technology, which plays a major role in the NAM model^13^ and in many other recent treatments of organizational and system learning.^12^, ^23^ Information technology creates new opportunities for rapidly gathering and synthesizing knowledge, assessing current performance, and providing feedback to managers and practitioners about effects of their actions.

The framework's distinction between leadership and culture contrasts with the NAM concept of “leadership instilled culture” (^13^, p. 18). Separating culture from leadership helps remind researchers to examine a broad range of factors besides leadership that may shape learning culture. Moreover, treating culture as a focal area for investigation may encourage assessment of ways that learning is affected by internal differentiation and fragmentation in beliefs, assumptions, and work routines.^54^, ^55^ The organizational change framework's focus on alignment among influential factors also contributes to understanding effects of external forces on organizational learning. For instance, payment incentives for care value, rather than for volume,^56^ may reinforce efforts of delivery organizations to learn how to deliver patient‐centered care and work with community services to promote population health. External incentives and policies will be more likely to foster organizational learning if they align with the care organizations' own strategies and goals for improving care.

Despite its utility, the organizational change framework also has limitations. One is insufficient attention to the impact of immediate operating conditions, as opposed to effects of broader forces in the political economy. Moreover, like the other two frameworks, the organizational change framework does not formally distinguish the roles of mid‐level management from those of executives and team leaders. Yet, middle managers often play critical roles in organizational learning^28^, ^57^ and change.^58^, ^59^

### 4.2 Multilevel framework of factors influencing organizational learning

**FIGURE 1:**
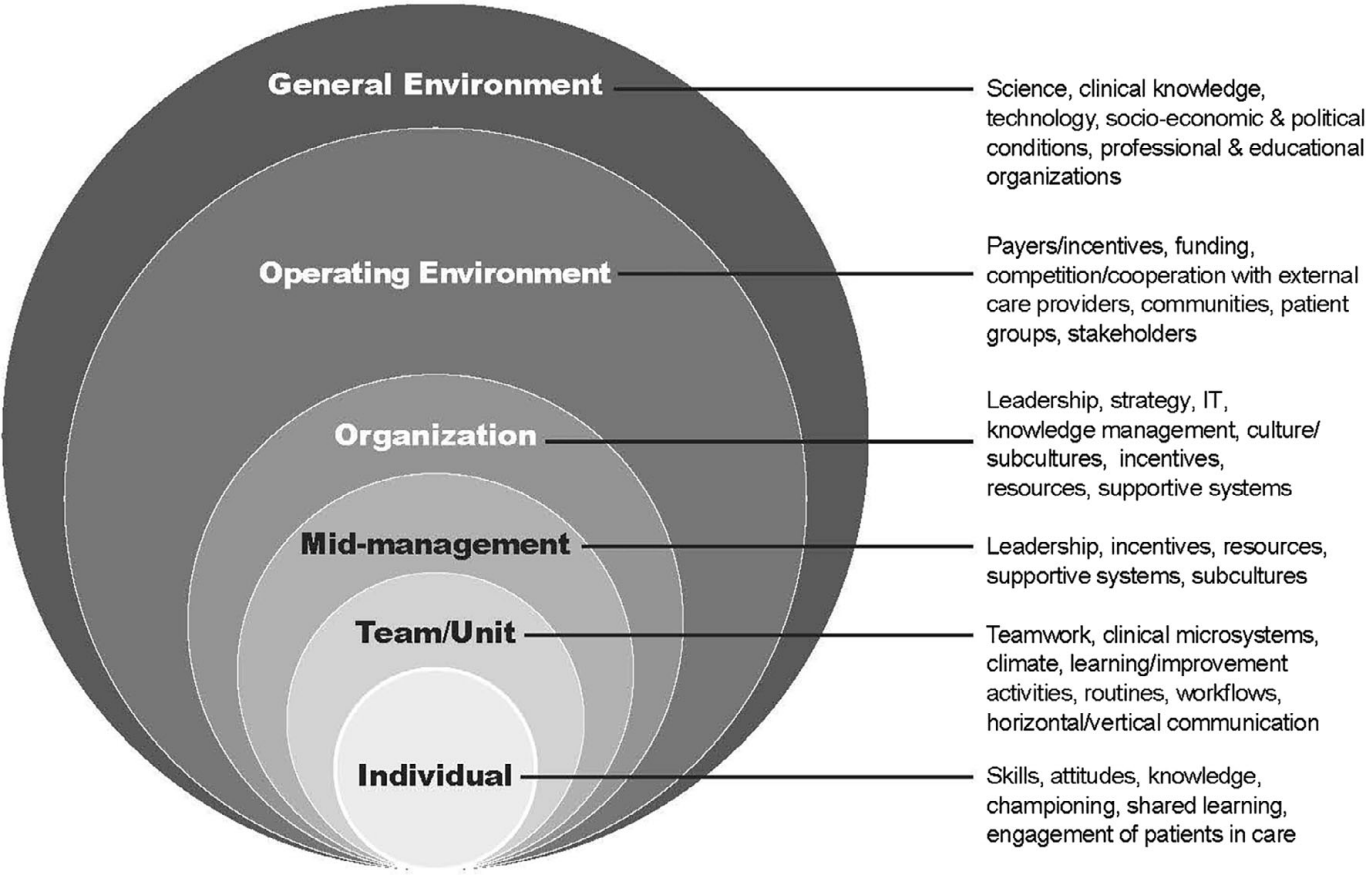
Factors influencing organizational learning

Figure 1 presents an integrative framework showing factors at multiple system levels that affect organizational learning. The framework incorporates many of the levels and factors identified in the above three frameworks and in research on organizational learning in health care (Figure 1, insert). Table 1 shows how the multilevel framework builds on and differs from the preceding frameworks (Table 1). The table lists some of the most relevant factors within the CFIR (“constructs) and the organizational change framework (“properties”). For brevity, the table omits relevant psychosocial factors in the socio‐ecological framework.

**TABLE 1:** Constructing the multilevel framework

Frameworks and relevant domains^a^				
Multilevel framework	CFIR	Socio‐ecological	Organizational change	Rationale for including level and/or domains in multilevel framework
General environment	Outer Setting (external policies and incentives; patient needs/resources)	System	Environment (political economy, markets, institutional forces)	Examine influences of slower‐to‐change, more distant environmental conditions (eg, policies, institutional norms, patient expectations).
Operating environment	Outer Setting (links to other organizations; peer pressure for implementation)	Community	Environment (incentives, relations to other organizations)	Distinguish immediate, more dynamic influences (eg, payment incentives, competitors, cooperating and allied organizations).
Organization	Inner Setting—service, unit, team levels. (structural characteristics, culture; learning climate; leadership engagement; resources; knowledge/information)	Organization	Overall organization (core properties: leadership, culture, team development, information technology)	Adopt widely used term; focus on total organization; emphasize alignment among core properties likely to have major effects on learning; distinguish leadership from culture.
Mid‐management	Not distinguished as separate level. Implementation Processes (planning, engaging; executing; reflecting and evaluating; actions of formal implementation leaders)	Not distinguished as level	Not included as separate level. (Core properties apply.	Focus on shared learning processes (vs change implementation); highlight strong influence of mid‐level managers.
Team/u	Inner Setting (variation across teams); (Implementation) Process (opinion leaders, formal leaders; champions, reflecting and evaluating)	Not included	Group/team (core properties)	Note how team processes affect organizational, as well as individual learning.
Individual	Involved Individuals (attributes; knowledge and beliefs about intervention; identification with organization; behavior)	Individual	Individual skills, motivation, behavior (eg, teamwork, technology use)	Indicate that individual learning may contribute to group learning; individuals can act as champions of organizational learning, engage patients in process/results of organizational learning.

Abbreviation: CFIR, Consolidated Framework for Implementation Research.

a CFIR domains are capitalized. CFIR constructs and organization change framework properties are in parentheses.

The multilevel framework, like its predecessors, anticipates interactions among influential factors within and across levels. Moreover, this new framework specially emphasizes impacts of alignment or misalignment among these factors. For example, the framework could lead researchers to ask whether influential factors, such as external and intraorganizational incentives, complement one another in encouraging LHS practices, or work at cross purposes. Similarly, the framework suggests that leaders seeking to foster learning systems consider whether their performance assessment and reward processes lead staff just to try to meet narrow performance standards or encourage questioning current practices, which can contribute to fundamental learning.^60^

The general environment in Figure 1 and Table 1 includes actors and conditions which may indirectly influence learning, including private and public decision makers and their policies and regulations; developers of scientific, clinical, and technical knowledge and innovations; social, economic, and political conditions; and activities of professional and educational organizations. The operating environment includes actors and conditions that can immediately and directly affect an organization's internal operations, including learning. Members of the operating environment include payers, external care providers, patients and community groups, suppliers, consulting organizations, entities directly overseeing care (eg, boards), and competitors. The organizational level includes a focal organization's goals, strategies, resources, and operational support for learning and care improvement; knowledge management and information technology capacities; culture; and performance assessment and reward system. In keeping with the importance of mid‐level leaders in system learning, Figure 1 includes a mid‐management level, which is not formally represented in the three frameworks just reviewed. The team/unit level draws attention to the many subgroups within health care organizations that may engage in group learning, such as those in nursing units, support departments, and primary care teams. The Individual level captures capacities and behavior of care providers and other staff that may affect shared learning.

The nesting of lower levels within higher ones in Figure 1 portrays the assumption that organizational learning like that envisioned in the NAM's LHS model^13^ will be more likely when characteristics fostering learning, such as supportive leadership, operate across multiple levels and align with one another. As suggested by the NAM, to ensure organizational learning based on scientific knowledge, care delivery organizations must develop their own internal mechanisms for gathering, managing, and implementing this external knowledge. Additionally, they need to develop technologies and processes that capture and use internal care experience to generate clinical knowledge. If, as advocated by the NAM, care delivery organizations are to learn from and with engaged patients, these organizations need to develop ties to the communities and patient groups they serve. The organizations will also benefit from developing clinical microsystems that place individual patients at the center of the care process and ensure shared decision‐making about care.^19^, ^52^ Furthermore, care delivery organizations will need to develop processes for translating learning from patients into internal knowledge and guidance for care givers. Additional factors, some of which are discussed later, must also be aligned across levels.

### 4.3 Multilevel analysis of learning

The value of this multilevel perspective on care organization learning can be illustrated by applying it to three, documented, improvement initiatives. The first of these began as a quality‐improvement intervention to help hospital intensive care units (ICUs) adhere to evidence‐based procedures for reducing catheter‐related infections.^61^ As the researchers observed the implementation process, they recognized the importance of factors beyond the boundaries of the participating ICUs, particularly organization‐level support from senior hospital managers for unit‐level implementation efforts. The research team gradually broadened their goals to encompass reshaping safety culture at the organization and mid‐management levels. They also developed hospital improvement collaboratives, thereby adding forces from the hospitals' operating environments to the repertoire of implementation strategies. These changes in program targets, goals, and implementation strategies reflected the researchers' developing, multilevel view of hospital learning to reduce preventable health‐acquired harms.^62^, ^63^ The researchers further extended their multilevel perspective in their “post hoc” examination of a successful, statewide infection‐control improvement collaborative in Michigan.^62^ They concluded that evidence‐based changes in team‐level practices were enhanced by peer pressure on infection control specialists from colleagues in other hospitals and by the examples that hospitals in the collaborative set for one another. These normative pressures, which originated in the hospitals' operating environment, encouraged hospital staff to share their performance data with other hospitals across the state and to strive to achieve the collaborative's agreed‐upon safety targets.^62^

The second initiative aimed to help four Australian hospitals adopt an ambitious quality program.^64^ The program, which embodied many principles of LHSs, sought to embed achievement of high quality care into leadership goals, operations, and hospital culture. During a 3‐year trial, one implementation hospital dropped out, while implementation in the other targeted hospitals proceeded more slowly and with less consistency than anticipated. Improvement in the implementing hospitals only occurred on one of eight quality metrics.^65^

The authors did not provide a multilevel analysis of their findings, but adopting this approach helped us identify and classify the main forces limiting the program's success. The most influential forces in the hospitals' operating environment were strict state standards for safety training and for reporting on clinical quality and safety. Hospital quality managers concentrated on compliance with these standards, thereby diverting their time and attention from the hospital quality improvement initiative.^66^ Shifts in other influential government policies diverted attention from the quality program, as did an accreditation review in one hospital. At the organization level, the hospitals' senior and mid‐level leaders reported additional challenges and priorities, which distracted them from dedication to the quality program. Furthermore, senior hospital leaders' concentration on compliance with state and federal regulations led them to underestimate the time and resources needed to plan and mobilize support for fundamental change in staff attitudes and behavior. At the unit and individual levels, clinicians reported insufficient support from immediate and higher level managers for far‐reaching behavioral and cultural change. A cross‐level interaction also created an implementation barrier: there was limited alignment between front‐line staff and managers about how to assure quality.

The third initiative involved development of a rheumatoid arthritis registry in Sweden, which was reported in a 15‐year retrospective study.^31^ As envisioned in the LHS model, care delivery organizations adopted new information technologies that became available in their general and operating environments. These technologies gradually supported use of the registry for clinical learning about medication effects. Within the delivery organizations, health information technology also enabled patient‐centered care—a learning system value. This approach to care benefitted from development of computerized procedures for patient entry of symptoms before visits; rapid calculation of a health status score; longitudinal plotting of symptoms and past treatments; and use of the data by individual practitioners and their patients in shared decision‐making about treatment options.

To provide a full multilevel account of the registry's contribution to system learning, it is necessary to locate the registry's development within the broader context of Sweden's electronic health records system.^67^ The registry's progress and learning contributions were substantially aided by alignment of supportive forces at the level of the nation, the regions (represented by County Councils), care organizations (hospitals and affiliated clinics), physicians, and patients. Sweden's Ministry of Health and Federation of County Councils created institutional foundations for collaborative information exchange across hospitals and physicians and paid for registry development and maintenance. Data collection and aggregation were aided by a national patient identifier and emerging foundations for national, interoperable data exchange. Individual hospitals shared data and bore data entry costs. Physicians specified data elements during the registry's development; recognized the contribution of registry data to clinical treatment; and demonstrated receptiveness to shared decision‐making. Patients, in turn, seem to have valued the richer and more consultative communication with their physicians, which was made possible by the electronic registry. Had physicians or patients approached registry use with different values and attitudes, the foundation for shared decision‐making might have been weaker. Had the registry lacked strong institutional support at the regional and national level, its developers would have had to follow a very different course. For example, in the United States, care systems and developers of new health information technologies face a more fragmented technical, financial, and regulatory environment. As a result, multiple, competing arthritis registries have emerged, each drawing on diverse sources of support and development, including professional associations, governmental and private funding agencies, industry, and hospitals.^68^

In summary, these three cases illustrate the analytic and practical value of applying a multilevel perspective to organizational learning. The infection control researchers gradually developed this type of perspective on their initiative and uncovered previously unreported ways in which professional peers and norms outside the hospitals affected internal learning at the team and organization levels. The papers that provided the basis for the other two examples did not apply a multilevel approach. Our reexamination of the findings in those papers points to the benefits of doing so. Multilevel framing can direct attention to potentially generalizable influences on system learning, such as the overemphasis in the Australian hospital quality initiative on meeting strict external standards for training and reporting.^65^ The reanalysis further suggests that system learning depends substantially on supportive interactions and alignments of influential factors within and across system levels.

### 4.4 Relations within and across levels

As these case studies suggest, to add precision to multilevel analysis, investigators should examine cross‐level relations among influential factors. Here we consider potential relations in three areas that organizational and implementation researchers have found to be particularly important for shared learning and improvement. Table 2 suggests research questions for each area. (Table 2). Similar questions could be developed for other types of influential factors.

**TABLE 2:** Research questions on leadership, incentives, and culture

Thematic area	Research questions
Incentives	How and to what extent do incentives at the operating environment, organization, mid‐management, and unit levels reinforce or undermine one another? How do care system leaders translate external incentives into internal directives and incentives for staff? Does this translation process encourage shared learning and experimentation or lead middle managers and staff to concentrate on meeting narrow targets? How do external and internal incentives influence collaboration for learning among organizations?
Culture	Are changes in societal norms, values, or patient expectations creating needs for organizational learning and change? How are care delivery organizations responding to these pressures? To what extent do prevailing organizational norms, values, and beliefs *within* a delivery system support or undermine receptiveness to shared learning? To what extent do assumptions and norms that senior leaders act on in practice (rather than simply espousing) reinforce or undermine transparency and willingness to suggest improvements among middle managers, clinicians, and other staff? Do similarities in values, taken‐for‐granted assumptions, and work routines across organizational divisions and functions provide foundations for collaborative examination of challenges, experimentation, and learning from experience—or do subcultural differences reduce opportunities for shared learning? How do differences in subcultures among medical specialties, occupations, nursing, staff, patients and their families and accepted ways of dealing with these differences impact organizational learning capacities?
Leadership	How do leaders scan and act on external knowledge and innovations that can contribute to learning and improvement? What practices and values do leaders at senior, mid, and unit levels notice, measure, reward, model, teach, and support? In what ways does this behavior foster and embed a culture of learning or block its development? To what degree do the strategies, goals, and behaviors of leaders align across levels and support learning and improvement? How do mid‐level managers mediate senior leaders' strategies? Do mid‐level managers integrate stakeholders; synthesize and diffuse information; champion and facilitate innovation?

#### 4.4.1 Incentives

External performance incentives in a delivery system's operating environment and their alignment with intraorganizational incentives, structures, and processes can substantially affect whether and how delivery systems learn to improve care quality and efficiency. For example, the growing wave of value‐based payment initiatives^7^, ^8^, ^69^ may encourage learning about care redesign. However, the potential and documented effects of these initiatives on organizational performance are not well established,^56^ and their effects on other forms of individual and organizational behavior are widely debated. Appropriate incentives may foster system learning by supporting activities such as research, professional education, care coordination, and provision of high value care.^29^ However, some analysts doubt whether current payment programs will foster better care outcomes or only produce undesired and unintended consequences, such as neglect of unmeasured or unrewarded practices; reduced intrinsic motivation^70^; and short cutting or cheating.^69^ Performance incentives may also reduce learning opportunities by discouraging collaboration within or between organizations^69^ and intensifying attention to short‐term results, thereby discouraging experimentation, innovation, and systematic evaluation of improvement programs.^71^ To investigate alignment among incentives and their impact on learning, researchers can pose questions like those in Table 2. For instance, they might investigate how external incentives are translated into internal sanctions and rewards and how these internal arrangements, in turn, affect individual, team, and organization‐level learning.

#### 4.4.2 Culture

Cultural influences on LHS processes and outcomes occur both within and outside of delivery organizations and systems. Gradual shifts in societal norms, values, and beliefs can create pressures and opportunities for delivery organizations to undertake learning and develop characteristics supporting learning. For example, growing recognition of the social determinants of health has led payers and delivery systems to seek ways to foster population health.^72^, ^73^ As they move in this direction, care providers and their leaders must engage in fundamental forms of organizational learning^60^—rethinking basic assumptions about their goals and modes of operation. Similarly, increasing expectations for transparency and public disclosure in healthcare encourage care delivery organizations to develop more outward facing organizational cultures.^74^ These cultures may encourage interorganizational collaboration and learning, rather than primarily seeking to satisfy internal stakeholders, such as boards and their professional leaders.

Many discussions of culture's intraorganizational influences on learning adopt a holistic, organization‐wide approach that concentrates on shared values, norms, beliefs, and assumptions that support learning and overcome learning barriers. For example, development of a culture of teamwork and standardized care promotes shared learning,^29^ as does the establishment of a culture promoting openness to new ideas, appreciation of differences, and psychological safety, which fosters transparency and sharing of insights and concerns.^18^, ^75^, ^76^ The holistic approach thus suggests research questions such as whether prevailing norms, values, and beliefs support or undermine share learning (see Table 2).

Besides examining effects of shared culture, analysts may gain insight by investigating divergence among cultural elements within care delivery systems or organizations.^54^ There may be cultural differences among organizations within a delivery system, along with differences within a particular organization across ranks, occupations (nurses vs physicians), divisions (eg, inpatient vs outpatient), clinical specialties, and operating units. Senior leaders, for example, may hold different beliefs about the value of experimentation and innovation than do department heads or front line staff.

Finally, investigators should closely examine implicit assumptions and behavior in practice. These can be distinguished from an organization's cultural symbols (eg, official slogans buzzwords and reward ceremonies) and its espoused norms and values (eg, vision statements).^77^ To explore culture in practice and effects of cultural divergence, researchers can pose questions like those shown in Table 2. For example, what implicit assumptions and norms about learning are expressed in leaders' behavior? How is learning affected by subcultural differences and prevailing ways of dealing with these differences?

#### 4.4.3 Leadership

Leaders help bridge between the organization and its general and operating environments.^30^, ^39^ In doing so, they can guide and support internal system learning.^13^, ^18^, ^29^ The multilevel framework encourages analysts to consider how governing boards and executives in care systems interpret and act on powerful forces in their general environment, such as the growing emphasis on wellness and the availability of personal digital technology. Similarly, it is important to examine how these leaders view and respond to key influences in the operating environment, such as consolidation among care delivery organizations. Then investigators can analyze how formulations of the system's vision, mission, and strategies may impact learning.^13^, ^14^, ^25^, ^77^ Within organizations, leaders at several organizational levels play a role in learning, as they put strategies into practice and influence implementation of new care practices^78^ and quality improvement.^37^ Leaders may synthesize and diffuse information and knowledge; help set mutually shared goals; establish and mediate strategic priorities; provide resources and training; empower front line staff by providing learning opportunities, listening to their inputs, and providing feedback; integrate stakeholders; champion and support innovation; and address external environmental challenges and opportunities.^18^, ^59^, ^79^, ^80^, ^81^ Hence, as suggested in Table 2, it is important for researchers to examine the practices and values that leaders at all levels attend to, reward, and support. Then researchers can assess the degree to which leaders' actions foster learning, align with one another, and align with other influential factors, such as incentives. Researchers may discern a wide range of processes through which mid‐level managers mediate senior leadership change strategies and sometimes fundamentally alter them.^80^ For example, the study of the Australian hospital quality program^65^ found that quality managers and other mid‐level managers reinterpreted program objectives articulated by hospital executives and by the state government. By doing so, they undermined opportunities to develop a culture of quality.

## 5 DISCUSSION

The multilevel perspective developed here and the framework in Figure 1 can serve as a guide for researchers as they examine learning within complex health care organizations; its diffusion throughout organizations; and its role in helping organizations achieve their immediate goals and meet long‐term challenges posed by external change. Wide variations in care quality and substantial waste in delivery of health care services,^82^ suggest the limits of relying on “one‐off,” first‐order solutions to pressing problems^75^ and underline the need for deep learning. Deep learning addresses the root causes of problems and questions underlying assumptions.^60^ It requires alignment and collaboration across multiple levels of organization and complex multilevel thinking.

The framework in Figure 1 may help investigators and practitioners broadly scan forces influencing improvement and learning and may point to otherwise unnoticed interactions among influential factors. Key factors and relations among them can be assessed for likely impact, theoretical importance, and amenability to change. After preliminary consideration of influential levels and key elements within them, researchers can select a subset of levels and factors for systematic data gathering and rigorous analysis. The multilevel framework can also be applied to the reporting of research findings. By specifying the levels, factors, and relations investigated in their studies, researchers can contribute to development of empirically grounded hypotheses for further testing and can facilitate synthesis of findings across studies.

Managers and practitioners may use the framework as a planning tool. For each level, they can consider requisite resources, incentives, training, skill mix, team structure, and time allocations that may be needed to promote and support learning. They can assess the cost and availability of these factors, how well they support one another, and how best to align them across organizational levels.

The NAM and other advocates of LHSs and learning organizations have articulated a set of complex and ambitious targets for transforming care delivery organizations and entire delivery systems. Research is needed that reveals the most critical paths toward developing and supporting the kind of learning envisioned by the LHS model. To that end, it would be valuable for researchers to unpack the complex multilevel, interactions of factors influencing learning within entire organizations and delivery systems.

## CONFLICT OF INTEREST

The authors affirm that they have no conflicts of interest associated with this article.

## Endnote

1 In 2015, the Institute of Medicine was renamed the National Academy of Medicine. We use the new name throughout this paper, even though the Institute of Medicine's foundational LHS reports appeared before the name change.
